# Immersion in water for pain relief and the risk of intrapartum transfer among low risk nulliparous women: secondary analysis of the Birthplace national prospective cohort study

**DOI:** 10.1186/1471-2393-14-60

**Published:** 2014-02-06

**Authors:** Mirjam Lukasse, Rachel Rowe, John Townend, Marian Knight, Jennifer Hollowell

**Affiliations:** 1 Department of Public Health and General Practice at the Faculty of Medicine, The Norwegian University of Science and Technology (NTNU), Håkon Jarls gate 11, N-7489 Trondheim, Norway; 2 Department of Health, Nutrition and Management, Faculty of Health Sciences, Oslo and Akershus University College of Applied Sciences, Postboks 364 Alnabru, N-0614 Oslo, Norway; 3 National Perinatal Epidemiology Unit, Nuffield Department of Population Health, University of Oxford, Old Road Campus, Oxford OX3 7LF, England

**Keywords:** Immersion in water, Midwifery-led care, Nulliparous women, Low risk, Pain management in labour, Intrapartum transfer, Intrapartum caesarean section

## Abstract

**Background:**

Immersion in water during labour is an important non-pharmacological method to manage labour pain, particularly in midwifery-led care settings where pharmacological methods are limited. This study investigates the association between immersion for pain relief and transfer before birth and other maternal outcomes.

**Methods:**

A prospective cohort study of 16,577 low risk nulliparous women planning birth at home, in a freestanding midwifery unit (FMU) or in an alongside midwifery unit (AMU) in England between April 2008 and April 2010.

**Results:**

Immersion in water for pain relief was common; 50% in planned home births, 54% in FMUs and 38% in AMUs. Immersion in water was associated with a lower risk of transfer before birth for births planned at home (adjusted RR 0.88; 95% CI 0.79–0.99), in FMUs (adjusted RR 0.59; 95% CI 0.50–0.70) and in AMUs (adjusted RR 0.78; 95% CI 0.69–0.88). For births planned in FMUs, immersion in water was associated with a lower risk of intrapartum caesarean section (RR 0.61; 95% CI 0.44–0.84) and a higher chance of a straightforward vaginal birth (RR 1.09; 95% CI 1.04–1.15). These beneficial effects were not seen in births planned at home or AMUs.

**Conclusions:**

Immersion of water for pain relief was associated with a significant reduction in risk of transfer before birth for nulliparous women. Overall, immersion in water was associated with fewer interventions during labour. The effect varied across birth settings with least effect in planned home births and a larger effect observed for planned FMU births.

## Background

Immersion in water during labour is a non-pharmacological method of managing labour pain that involves the pregnant woman’s abdomen being completely submerged in warm water [1]. It requires a tub, bath or pool which is larger than an average domestic bath [1]. This method of pain relief was widely introduced in maternity care across the western world during the nineties [2-7]. It is most widely used in midwifery-led settings, (36-47% of ‘low risk’ births in midwifery-led settings vs. 9% in obstetric units) [8] although the majority of obstetric units have a ‘birthing pool’ [9]. In the UK, midwifery-led care may be offered to healthy women with low risk pregnancies and can take place at a woman’s home, in a freestanding midwifery unit (FMU), situated on a site geographically separate from a consultant-led obstetric unit (OU), or in an alongside midwifery unit (AMU), situated in the same building or on the same site as an OU [10]. Women are transferred to an OU if they require medical, anaesthetic or obstetric care. Transfer from midwifery-led care is common (21-26% of births planned in non-OU settings), especially for first-time mothers [7,11].

A Cochrane systematic review of immersion in water in labour and birth found that immersion in water significantly reduced the duration of the first stage of labour and reduced the use of epidural anaesthesia, but did not significantly reduce intrapartum caesarean section in the included RCTs [1]. A large observational study of UK births in obstetric units, midwifery units and home settings [7] however, found low levels of intrapartum caesarean section in women who used a birthing pool. Intrapartum transfer rates from home and FMUs were also low in this study compared with those observed in the Birthplace prospective cohort study, which assessed intrapartum outcomes by planned place of birth [7,12]. These findings suggest that the use of immersion in water for pain relief may be associated with a lower risk of intrapartum transfer and possibly a reduction in other interventions and adverse outcomes. Intrapartum transfer and intervention rates are highest in nulliparous women so this group is of particular interest. The aim of the study reported here was to assess whether immersion in water for pain relief in labour is associated with a lower risk of intrapartum transfer and other intrapartum interventions and adverse maternal outcomes in low risk nulliparous women planning birth outside an obstetric unit.

## Methods

### Source of data and study population

This study used data from the Birthplace in England national prospective cohort study, which was designed to compare perinatal and maternal outcomes and interventions by planned place of birth at the start of care in labour in England [12].

The cohort study methods are described in full elsewhere [8,12]. Briefly, the Birthplace cohort included a total of 79,774 births between April 2008 and April 2010, including 32,257 planned OU births from a stratified random sample of 36 OUs, 11,666 planned births in 53 freestanding midwifery units (FMUs), 17,582 planned births in 43 alongside midwifery units (AMUs) and 18,269 planned home births from 142 NHS trusts across England. Births were eligible for inclusion if the woman was planning a vaginal birth and received some labour care from an NHS midwife in her planned birth setting. Women who had an elective caesarean section or caesarean section before the onset of labour, presented in preterm labour (<37 weeks’ gestation), had a multiple pregnancy, or who were “unbooked” (received no antenatal care) or had an unplanned home birth were excluded. Stillbirths occurring before the start of care in labour were excluded. Women were defined as ‘low risk’ if, prior to the onset of labour, they were not known to have any of the medical or obstetric risk factors listed in the NICE intrapartum guidelines [13].

Data were recorded by the midwife attending the birth using a study-specific data collection form started during labour and completed on or after the fifth postnatal day. Where a woman transferred to another unit, the form transferred with the woman and data collection was continued in the receiving unit. Data collected for all women included maternal characteristics, medical or obstetric risk factors known prior to the onset of labour, complicating conditions identified by the midwife at the start of care in labour, whether the woman used immersion in water for pain relief at any time during labour, obstetric interventions, including labour augmentation with oxytocin, epidural or spinal analgesia, forceps or ventouse delivery, intrapartum caesarean section, and maternal and neonatal outcomes, including 3^rd^/4^th^ degree tear and blood transfusion for the mother and Apgar score at 5 minutes and admission to a neonatal unit for the baby. Where a woman was transferred during labour or immediately after the birth, information was recorded about the primary reason for transfer and about the timing of the transfer, including the time of the decision to transfer and the time that the transfer started.

Information was not collected about the urgency of the transfers so we used an approach adopted for another study [14] to classify transfers as being for ‘potentially urgent reasons’ when the primary reason for transfer was antepartum haemorrhage, failure to progress in the second stage and fetal distress in the first or second stage. This method of classification identifies women with an increased risk of instrumental delivery, caesarean section or adverse neonatal outcome within an hour of arrival in the OU [14].

The study population for the analyses reported here was ‘low risk’ nulliparous women in the Birthplace cohort with a term pregnancy (37-42^+0^ weeks’ gestation) who planned to give birth in a non-obstetric unit setting and who did not have ‘complicating conditions’ at the start of labour care that might either contraindicate immersion in water or be a reason for transfer to an obstetric unit [11]. Women with the following ‘complicating conditions’ noted by the midwife at the start of care in labour were excluded: meconium stained liquor, prolonged rupture of membranes (>18 hours), hypertension (diastolic ≥90 mmHg more than once, 20 min. apart, and ≥100 mmHg once of systolic ≥ 160 mmHg at least once), abnormal vaginal bleeding, abnormal fetal heart rate, or non-cephalic presentation. Finally, we excluded women with missing data on use of water immersion to relieve labour pain.

### Outcomes

The main outcomes were:

• Intrapartum transfer before birth

• Intrapartum caesarean section

• Straightforward vaginal birth, defined as birth without forceps, ventouse or caesarean, with no 3^rd^ or 4^th^ degree tear and no blood transfusion.

Secondary outcomes were:

• Transfer for failure to progress in the first stage of labour

• Transfer before birth for ‘potentially urgent reasons’

•Transfer for pain relief or epidural analgesia

• Augmentation in labour with oxytocin

• Epidural or spinal analgesia

### Statistical analysis

To assess the association between immersion in water for pain relief and our outcome measures we used log Poisson regression to calculate relative risks (RR) and 95% confidence intervals (CI), both unadjusted and adjusted for maternal characteristics (maternal age, ethnic group, understanding of English, marital or partner status, index of multiple deprivation (IMD) score [15], and gestational age at birth). Analyses were performed separately for each type of birth setting. As in previous analyses of the Birthplace cohort, probability weights were used to adjust for the varying duration of each unit or trust’s participation in the study and robust variance estimation was used to allow for the clustered nature of the data within maternity units and NHS trusts (for home births). For each outcome, we report the number of events, the number of births, the weighted incidence and the unadjusted and adjusted relative risks. For completeness, we also estimated the unadjusted relative risks restricted to births included in the adjusted analysis, but because there was a low level of missing data this had a minimal effect on the estimated RRs and CIs so these are not reported. Women not using immersion in water for pain relief were the reference category in all analyses. To assess whether the associations between immersion in water for pain relief and our main outcomes differed by planned place of birth, a Wald test for statistical interaction was performed. Where the interaction was not significant at the 5% level, an analysis to estimate the effect of water immersion adjusting for the effect of planned place of birth, but ignoring the interaction between planned place of birth and water immersion, was conducted.

Post protocol we decided to perform two additional statistical analyses. First, because transfer, or birth occurring soon after the start of care in labour might reduce a woman’s opportunity to use immersion in water, and this could potentially confound the relationship between immersion in water and our study outcomes, we carried out a sensitivity analysis in which we repeated the main analysis, but excluded women for whom the decision to transfer was taken within the first 90 minutes after the start of labour care, who gave birth within 90 minutes, or where the timing of either of these events was unknown. Second, to check that there were no obvious adverse effects on neonatal outcomes associated with immersion in water for pain relief, we estimated the weighted proportion, and unadjusted and adjusted relative risks of Apgar score below 7 at 5 minutes and neonatal admission, by planned place of birth for women who did and did not use immersion in water for pain relief. Stata version 11.2 was used for all analyses [16].

### Ethical approval

Research ethics committee approval for the Birthplace study was obtained from the Berkshire Research Ethics Committee and did not require consent to be sought from participants as no personally identifiable data were collected (MREC ref 07/H0505/151).

## Results

The Birthplace cohort contained 17,917 ‘low risk’ nulliparous women planning birth at home, in an AMU or in an FMU (see Additional file 1: Figure S1 for flow chart). Of these, 1,215 (7%) were excluded because they had ‘complicating conditions’ at the start of care in labour that were considered to be contraindications to immersion in water; and 125 (<1%) were excluded because data on use of immersion in water was missing.

The study sample consisted of 16,577 ‘low risk’ nulliparous women, 7,733 planning AMU birth, 4,831 planning FMU birth, and 4,013 planning birth at home. Immersion in water for pain relief was common: 50% in planned home births, 54% in FMUs and 38% in AMUs. The characteristics of women in the study sample are presented in Table 1. Women under 25 were less likely to use water immersion for pain relief across all planned places of birth while women aged 30–34 were more likely to use water immersion for pain relief with the same pattern apparent for all planned places of birth. Across all midwifery-led planned places of birth women who were not fluent in understanding English were less likely to use water immersion for pain relief, as were women living in more deprived areas and women who were single or unsupported by their partner.

**Table 1:** Characteristics of low risk women and babies by planned place of birth and immersion in water for pain relief

	**Home**				**FMU**				**AMU**			
	**n = 4013**				**n = 4831**				**n = 7733**			
	**No immersion**		**Immersion**		**No immersion**		**Immersion**		**No immersion**		**Immersion**	
	**n = 2002**		**n = 2011**		**n = 2216**		**n = 2615**		**n = 4807**		**n = 2926**	
	**n**	**%**^**1**^	**n**	**%**^**1**^	**n**	**%**^**1**^	**n**	**%**^**1**^	**n**	**%**	**n**	**%**^**1**^
**Maternal age**												
Mean (SD)	29.7	(5.3)	30.6	(4.7)	26.2	(5.6)	27.6	(5.7)	26.5	(5.6)	27.5	(5.6)
Under 20	87	4.3	30	1.5	297	13.4	248	9.5	566	11.8	272	9.3
20–24	241	12.0	159	7.9	609	27.5	554	21.2	1300	27.0	626	21.4
25–29	607	30.3	611	30.4	659	29.7	783	29.9	1465	30.5	901	30.8
30–34	675	33.7	808	40.2	474	21.4	735	28.1	1048	21.8	795	27.2
35–39	350	17.5	362	18.0	161	7.3	261	10.0	391	8.1	303	10.4
40+	39	1.9	37	1.8	12	0.5	32	1.2	29	0.6	22	0.8
Missing	3	0.1	4	0.2	4	0.2	2	0.1	8	0.2	7	0.2
**Ethnicity**												
White	1899	94.9	1898	94.4	2030	91.6	2428	92.8	3876	80.6	2548	87.1
Non-white	102	5.1	108	5.4	186	8.4	186	7.1	919	19.1	369	12.6
Missing	1	0.0	5	0.2	0	0.0	1	0.04	12	0.3	9	0.3
**Understanding English**												
Fluent	1983	99.1	2004	99.7	2111	95.3	2563	98.0	4320	89.9	2755	94.2
Not fluent	17	0.8	4	0.2	96	4.3	52	2.0	471	9.8	159	5.4
Missing	2	0.1	3	0.1	9	0.4	0	0.0	16	0.3	12	0.4
**Marital/Partner status**												
Married/Living together	1881	94.0	1924	95.7	1913	86.3	2380	91.0	4111	85.5	2587	88.4
Single/unsupported by partner	110	5.5	75	3.7	276	12.5	208	8.0	638	13.3	282	9.6
Missing	11	0.5	12	0.6	27	1.2	27	1.1	58	1.2	57	1.9
**Body mass index (kg/m**^**2**^**)**												
Not recorded	391	19.5	371	18.4	334	15.1	484	18.5	810	16.8	509	17.4
Less than 18.5	32	1.6	42	2.1	60	2.7	51	2.0	138	2.9	87	3.0
18.5–24.9	1052	52.6	1077	53.6	1159	52.3	1412	54.0	2505	52.1	1615	55.2
25.0–29.9	406	20.3	403	20.0	502	22.7	514	19.7	1019	21.2	551	18.8
30.0–35.0	109	5.4	108	5.4	157	7.1	153	5.9	321	6.7	158	5.4
Missing	12	0.6	10	0.5	4	0.2	1	0.0	14	0.3	6	0.2
**Index of Multiple Deprivation (IMD) quintiles**												
1^st^ Least deprived	412	20.6	427	21.2	400	18.1	606	23.2	693	14.4	476	16.3
2^nd^	408	20.4	432	21.5	475	21.4	630	24.1	733	15.2	524	17.9
3^rd^	454	22.7	455	22.6	464	20.9	561	21.5	921	19.2	628	21.5
4^th^	403	20.1	426	21.2	478	21.6	422	16.1	1151	23.9	690	23.6
5^th^ Most deprived	314	15.7	259	12.9	392	17.7	389	14.9	1294	26.9	602	20.6
Missing	11	0.5	12	0.6	7	0.3	7	0.3	15	0.3	6	0.2
**Gestation (completed weeks)**												
Mean (SD)	39.7	(1.1)	39.8	(1.0)	39.7	(1.1)	39.8	(1.0)	39.7	(1.1)	39.8	(1.0)
37	53	2.6	37	1.8	70	3.2	68	2.6	153	3.2	79	2.7
38	219	10.9	174	8.7	209	9.4	226	8.6	475	9.9	272	9.3
39	491	24.5	430	21.4	536	24.2	554	21.2	1179	24.5	688	23.5
40	739	36.9	792	39.4	833	37.6	1019	39.0	1833	38.1	1129	38.6
41	480	24.0	546	27.2	557	25.1	729	27.9	1134	23.6	729	24.9
42^+0^	20	1.0	32	1.6	11	0.5	19	0.7	33	0.7	29	1.0
**Birth weight (grams)**												
mean (SD)	3452	(419)	3497	(430)	3391	(419)	3441	(417)	3380	(423)	3447	(412)
Less than 2500 g	15	0.7	16	0.8	30	1.4	21	0.8	74	1.5	22	0.8
2500-2999 g	259	12.9	217	10.8	345	15.6	325	12.4	788	16.4	351	12.0
3000-3499 g	810	40.5	756	37.6	968	43.7	1138	43.5	2090	43.5	1247	42.6
3500-3999 g	702	35.1	774	38.5	698	31.5	873	33.4	1465	30.5	1009	34.5
4000-4499 g	197	9.8	210	10.4	161	7.3	229	8.8	350	7.3	267	9.1
≥4500 g	13	0.6	33	1.6	14	0.6	29	1.1	28	0.6	20	0.7
Missing	6	0.3	5	0.2	0	0.0	0	0.0	12	0.2	10	0.3

^1^Unweighted and unadjusted, percentages may not add to 100 due to rounding.

Immersion in water for pain relief was associated with a significantly lower relative risk (adjusted) of transfer before birth across all birth settings, ranging from a 41% reduction (adjusted RR 0.59, 95% CI 0.50 - 0.70) in planned FMU births to 11% (adjusted RR 0.88, 95% CI 0.79-0.99) in planned home births (Table 2). The effect of immersion in water varied by planned birth setting (Wald test p < 0.001) so we did not estimate a pooled relative risk across settings.

**Table 2:** Association between immersion in water and main study outcomes by planned place of birth

	**Events**	**Births**	**Weighted**^**1**^		**Unadjusted**^**1**^		**Adjusted**^**1,2**^	
	**n**	**n**	**%**	**(95% CI)**	**RR**	**(95% CI)**	**RR**	**(95% CI)**
**Transfer before birth**								
**Home**								
No immersion	661	1977	32.8	(30.0–35.7)	1	-	1	-
Immersion	625	1999	30.3	(27.9–32.9)	0.93	(0.83–1.03)	0.88	(0.79–0.99)
**FMU**								
No immersion	747	2200	31.5	(27.3–36.1)	1	-	1	-
Immersion	590	2594	20.3	(17.2–23.8)	0.64	(0.54–0.76)	0.59	(0.50–0.70)
**AMU**								
No immersion	1657	4749	34.5	(31.3–37.8)	1	-	1	-
Immersion	828	2889	28.7	(25.0–32.8)	0.83	(0.73–0.96)	0.78	(0.69–0.88)
**No pooled analysis as Wald test for interaction was p = <.001**								
**Intrapartum caesarean section**								
**Home**								
No immersion	150	2000	7.8	(6.1–10.0)	1	-	1	-
Immersion	146	2010	7.5	(6.2–9.1)	0.96	(0.73–1.27)	0.84	(0.63–1.11)
**FMU**								
No immersion	170	2216	7.1	(5.4–9.2)	1	-	1	-
Immersion	133	2615	5.0	(4.2–6.0)	0.71	(0.52–0.96)	0.61	(0.44–0.84)
**AMU**								
No immersion	356	4801	7.2	(6.1–8.5)	1	-	1	-
Immersion	195	2922	6.9	(5.4–8.7)	0.95	(0.74–1.24)	0.87	(0.67–1.13)
**Combined home, FMU and AMU (Wald test p = 0.179)**								
No immersion	676	9017	7.3	(6.4–8.2)	1	-	1	-
Immersion	474	7547	6.5	(5.6–7.5)	0.90	(0.75–1.08)	0.80	(0.67–0.97)
**Straightforward vaginal birth**^**3**^								
**Home**								
No immersion	1521	1985	76.5	(73.6–79.3)	1	-	1	-
Immersion	1517	1999	76.2	(73.8–78.4)	1.00	(0.95–1.04)	1.02	(0.97–1.07)
**FMU**								
No immersion	1665	2209	77.2	(73.2–80.8)	1	-	1	-
Immersion	2121	2607	82.3	(79.8–84.5)	1.07	(1.01–1.12)	1.09	(1.04–1.15)
**AMU**								
No immersion	3421	4749	72.3	(69.4–75.0)	1	-	1	-
Immersion	2145	2886	73.0	(68.9–76.8)	1.01	(0.95–1.07)	1.05	(0.99–1.10)
**Combined home, FMU and AMU (Wald test p = 0.078)**								
No immersion	6607	8943	73.7	(71.5–75.7)	1	-	1	-
Immersion	5783	7492	76.1	(73.5–78.5)	1.02	(0.98–1.06)	1.05	(1.02–1.09)

^1^Weighted to adjust for clustering and each unit’s duration of participation.

^2^Adjusted for maternal age, ethnic group, understanding of English, marital/partner status, index of multiple deprivation score quintile, gestation (completed weeks), and planned place of birth in the pooled analysis.

^3^The positive risk of a straightforward vaginal birth.

Analyses by planned place of birth showed that immersion in water for pain relief was associated with a lower risk of intrapartum caesarean section in all settings after adjustment for maternal characteristics (Table 2). Although this association was statistically significant in FMUs only (adjusted RR 0.61, 95% CI 0.44–0.84), there was no evidence that the effect of immersion differed significantly by planned birth setting (Wald test p = 0.179) and pooled analysis showed that, overall, immersion in water for pain relief was associated with a 20% reduction in the risk of intrapartum caesarean section (adjusted pooled RR 0.80, 95% CI 0.67–0.97).

Immersion in water was significantly associated with a higher chance of a straightforward vaginal birth in FMUs (Table 2). The chance of a straightforward vaginal birth was not significantly higher in planned home or AMU births when immersion in water was used, but the effect of immersion in water did not differ significantly by planned place of birth (Wald test p = 0.078) and in the pooled analysis the adjusted relative risk of a straightforward vaginal birth for the three birth settings combined was 1.05 (95% CI 1.02–1.09).

We did not observe significant differences in the risk of transfer for failure to progress in the first stage between women who had and had not used immersion in water in any of the settings (Table 3). Transfers for potentially urgent reasons occurred significantly less often (RR 0.61, 95% CI 0.48–0.77) in women who used immersion in water in planned FMU births, but not in the other two settings (Table 3). Immersion in water was associated with a significantly lower risk of transfer for epidural or other pain relief for births planned at FMUs and AMUs but not at home (Table 3). The same pattern of associations was seen for immersion in water and subsequent use of augmentation with oxytocin and epidural or spinal analgesia after transfer (Table 3).

**Table 3:** Association between immersion in water and secondary outcomes by planned place of birth

	**Events**	**Births**	**Weighted**^**1**^		**Unadjusted**^**1**^		**Adjusted**^**1,2**^	
	**n**	**n**	**%**	**(95% CI)**	**RR**	**(95% CI)**	**RR**	**(95% CI)**
**Transfer for failure to progress in 1st stage of labour**								
**Home**								
No immersion	236	1997	11.0	(9.4–12.9)	1	-	1	-
Immersion	224	2004	10.9	(9.6–12.5)	0.99	(0.81–1.22)	0.94	(0.77–1.16)
**FMU**								
No immersion	212	2203	8.8	(7.1–10.8)	1	-	1	-
Immersion	216	2603	7.5	(6.2–9.0)	0.85	(0.67–1.06)	0.80	(0.63–1.02)
**AMU**								
No immersion	410	4756	8.5	(6.7-10.6)	1	-	1	-
Immersion	250	2900	8.4	(7.0–10.1)	0.99	(0.77–1-27)	0.98	(0.78–1.23)
**Transfer for ‘potentially urgent reasons’**								
**Home**								
No immersion	191	1997	9.5	(7.9–11.2)	1	-	1	-
Immersion	210	2004	10.4	(9.1–11.9)	1.10	(0.89–1.36)	1.06	(0.86–1.30)
**FMU**								
No immersion	274	2203	11.7	(9.9–13.8)	1	-	1	-
Immersion	228	2603	7.8	(6.3–9.6)	0.67	(0.53–0.84)	0.61	(0.48–0.77)
**AMU**								
No immersion	587	4756	12.0	(10.4–13.7)	1	-	1	-
Immersion	338	2900	11.5	(9.6–13.8)	0.97	(0.79–1.19)	0.91	(0.75–1.09)
**Transfer for epidural or other pain relief**								
**Home**								
No immersion	86	1997	4.3	(3.4–5.6)	1	-	1	-
Immersion	87	2004	4.1	(3.2–5.2)	0.94	(0.69–1.28)	0.88	(0.63–1.22)
**FMU**								
No immersion	73	2203	3.0	(2.0–4.6)	1	-	1	-
Immersion	64	2603	2.1	(1.5–2.9)	0.68	(0.46–1.01)	0.63	(0.42–0.94)
**AMU**								
No immersion	297	4756	6.8	(5.6–8.2)	1	-	1	-
Immersion	127	2900	5.0	(4.0–6.3)	0.73	(0.59–0.92)	0.64	(0.51–0.78)
**Augmentation with oxytocin**								
**Home**								
No immersion	321	1991	15.1	(12.9–17.5)	1	-	1	-
Immersion	329	2002	16.0	(14.4–17.8)	1.07	(0.88–1.29)	1.00	(0.82–1.22)
**FMU**								
No immersion	371	2204	15.2	(12.5–18.5)	1	-	1	-
Immersion	315	2602	10.8	(9.3–12.5)	0.71	(0.58–0.87)	0.64	(0.52–0.79)
**AMU**								
No immersion	871	4791	17.7	(15.7–19.8)	1	-	1	-
Immersion	453	2913	15.9	(13.5–18.6)	0.90	(0.75–1.08)	0.84	(0.71–0.99)
**Epidural or spinal analgesia**								
**Home**								
No immersion	418	1993	20.5	(18.0–23.3)	1	-	1	-
Immersion	440	2003	21.5	(19.3–23.8)	1.05	(0.90–1.22)	0.97	(0.83–1.14)
**FMU**								
No immersion	467	2208	20.0	(17.0–23.4)	1	-	1	-
Immersion	439	2607	15.9	(13.6–18.5)	0.80	(0.65–0.97)	0.72	(0.58–0.89)
**AMU**								
No immersion	1149	4793	24.1	(21.6–26.8)	1	-	1	-
Immersion	632	2915	22.7	(19.5–26.3)	0.94	(0.81–1.10)	0.87	(0.75–1.00)

^1^Weighted to adjust for clustering and each unit’s duration of participation.

^2^Adjusted for maternal age, ethnic group, understanding of English, marital/partner status, index of multiple deprivation score quintile, and gestation (completed weeks).

Table 4 describes the primary reasons for decision to transfer by planned place of birth and immersion in water. The proportion of transfers for failure to progress in the second stage was slightly higher in the immersion in water groups in all settings.

**Table 4:** Primary reason for decision to transfer by use of water immersion and by planned place of birth

**Reasons for decision to transfer**	**Home n = 4013**				**FMU n = 4831**				**AMU n = 7733**			
	**No immersion**		**Immersion**		**No immersion**		**Immersion**		**No immersion**		**Immersion**	
	**n = 2002**		**n = 2011**		**n = 2216**		**n = 2615**		**n = 4807**		**n = 2926**	
	**n**	**%**^**1**^	**n**	**%**^**1**^	**n**	**%**^**1**^	**n**	**%**^**1**^	**n**	**%**^**1**^	**n**	**%**^**1**^
Not transferred	1158	58.7	1159	58.7	1339	62.6	1826	71.5	2847	59.3	1897	64.7
‘Antepartum reasons’*												
Malposition	3	0.1	2	0.1	4	0.1	4	0.1	15	0.2	7	0.2
Malpresentation	13	0.6	6	0.3	13	0.5	2	0.1	24	0.4	5	0.2
Failure to progress (1^st^ stage)	236	11.0	224	10.9	212	8.8	216	7.4	410	8.3	250	8.3
Fetal distress (1^st^ stage)	45	2.4	28	1.4	97	4.5	38	1.5	154	3.1	47	1.8
Meconium staining	102	5.3	71	3.2	133	5.5	72	2.6	236	4.8	88	2.8
Epidural request	64	3.1	61	2.9	69	2.9	64	2.1	296	6.7	124	4.8
Hypertension	8	0.4	7	0.3	18	1.0	4	0.1	32	0.6	5	0.4
Pain relief	22	1.2	26	1.2	4	0.1	0	-	1	<0.1	3	0.1
Antepartum haemorrhage	15	0.7	7	0.4	20	0.9	5	0.2	39	0.8	11	0.4
Failure to progress (2^nd^ stage)	113	5.5	158	7.8	137	5.3	167	5.5	322	6.5	235	7.7
Fetal distress (2^nd^ stage)	15	0.7	13	0.6	12	0.6	15	0.5	62	1.2	41	1.4
Other maternal	22	1.1	22	1.0	13	0.6	5	0.2	49	1.0	13	0.4
Other fetal	7	0.4	6	0.3	8	0.4	5	0.2	14	0.3	5	0.2
Other	11	0.5	5	0.3	9	0.3	2	0.1	15	0.2	8	0.3
Reason not recorded	2	0.1	3	0.2	10	0.5	4	0.1	29	0.9	11	0.3
**Postpartum transfers**												
Postpartum haemorrhage	26	1.3	24	1.2	10	0.5	25	1.0	29	0.9	22	0.8
Retained placenta	29	1.4	47	2.3	36	1.8	41	1.6	53	1.0	37	1.1
Repair of perineal trauma	86	4.2	113	5.4	55	2.4	84	3.4	156	3.1	97	3.5
Other maternal	5	0.2	5	0.2	2	0.1	8	0.2	1	<0.1	4	0.1
Neonatal concerns	16	0.8	20	0.9	11	0.5	20	0.8	1	<0.1	1	0.1
Other	1	<0.1	3	0.2	1	<0.1	1	<0.1	10	0.2	4	0.2
Transferred (timing & reason not known)	3	0.2	1	0.1	3	0.1	7	0.9	12	0.4	11	0.4

^1^Weighted percent.

*13 transfers for ‘antepartum reasons ‘occurred after the birth (9 for ‘meconium staining’ and 5 for ‘fetal distress, 2^nd^ stage’).

Restriction of the analysis to women who were still planning to give birth in their initial planned setting 90 minutes after their start of care in labour did not substantively affect the estimates of the relative risk of transfer before birth (Additional file 2: Table S1).

The weighted but unadjusted incidence of transfer for neonatal concerns was slightly higher for the immersion in water groups from home or FMU (Table 4), but immersion in water was not associated with an increased risk of Apgar score below 7 at 5 minutes or neonatal admission in any setting (Additional file 3: Table S2).

## Discussion

### Main findings

The aim of the study was to assess whether immersion in water for pain relief in labour was associated with a lower risk of intrapartum transfer and other intrapartum interventions and adverse maternal outcomes in low risk nulliparous women planning birth outside an obstetric unit. We found that immersion in water for pain relief was associated with fewer transfers before birth in all settings, and in some settings with better maternal outcomes in the form of fewer intrapartum caesarean sections, an increase in straightforward vaginal births and fewer interventions such as epidural analgesia and augmentation with oxytocin. A consistent beneficial effect of immersion in water was seen across all outcomes considered for births planned in freestanding midwifery units. The positive effect of water immersion was also seen for births planned in alongside midwifery units, although the effect was not statistically significant for all outcomes. Water immersion was not associated with a lower risk of caesarean section or a lower risk of other interventions in births planned at home.

### Strengths and weaknesses of the study

The data for this study were of high quality, prospectively collected from a nationally representative sample of birth planned in AMUs, FMUs and at home. An important strength is that we were able to study a homogeneous, low risk population and to control for a number of maternal characteristics that might confound the relationship between immersion in water and the outcomes studied. However, the women who used immersion in water were a self-selected group and we cannot rule out the possibility that some of the observed associations may be due to unmeasured differences in the characteristics of women who did and did not use immersion in water for pain relief. It is possible, for example, that women who choose immersion in water for pain relief may have a stronger preference for a birth without intervention than those who do not and this may affect decisions about transfer taken during labour and subsequent interventions. For example, women’s willingness to accept obstetric intervention was found in one study to be a significant predictor of use of epidural analgesia [17].

A further strength of the study is that we were able to analyse outcomes separately in different birth settings and, in particular, the uniquely large sample size of planned home births allowed us to study them separately from the other planned places of birth. The large UK birthing pool study collected data from only 155 planned home births and analysed their outcome together with births planned in freestanding midwifery units [7].

Because the Birthplace study was designed to address questions relating to the safety of planned place of birth, however, only limited data of relevance to our research question were collected. For example, we lacked information on the duration of immersion in water, at what stage during labour they entered the water [6], the temperature of the water [18], the size and type of pool used [19], or whether the birth took place in water [5], all factors which may influence the impact of water immersion.

### Comparison with other studies

Our study showed lower transfer rates in women who used immersion in water for pain relief. Besides alleviating pain and thus reducing the need for epidural analgesia, it has been suggested that immersion in water increases relaxation, reduces blood pressure, shortens labour, and empowers women [20]. All these factors may contribute to the lower transfer rate seen in this group.

The transfer rates observed in the immersion groups in our study were comparable to those in the birthing pool study for transfers before birth, although overall transfer rates, including postpartum transfers, appeared to be slightly higher in our study [7]. We found that 28.5% of the nulliparous women who used immersion in water were transferred from an FMU to an OU compared with 20% of nulliparous women in the ‘community’ setting in the birthing pool study [7]. The proportions of nulliparous women using the birth pool who were transferred from an AMU to an OU were also broadly similar: 35.3% in our study compared with 31% in the birthing pool study [7].

In contrast to the results of the RCTs included in the Cochrane review we observed a lower risk of intrapartum caesarean section associated with immersion in water, but only for births planned in an FMU [1]. Our study’s intrapartum caesarean section rates for women who used immersion in water were similar to or slightly higher than those seen in the birthing pool study (5% vs. 3.1% respectively for planned FMU/community births and 6.9% vs. 6.1% for planned AMU births) [7]. Consistent with the Cochrane review we found a significant reduction in the epidural rate amongst women who used immersion in water in FMUs and AMUs [1].

Our findings showed that immersion in water had limited effect on transfer and no significant effect on intrapartum interventions for births planned at home. The lack of the positive impact of immersion in water in births planned at home compared to those planned in midwifery units was surprising and has not been reported before. There are several possible explanations for this finding. First, women who plan a birth at home may be different from women choosing to give birth at an FMU or AMU, resulting in selection bias. Second, the type of pool used at home may be smaller with less room for full immersion and freedom of movement [19,21]. Third, some of the benefits observed for immersion in water may be due to other components of care, such as the support of caregivers who have a similar philosophy about childbirth and consistent advice about when, during labour, to use the pool [1]. Evidence suggests that early immersion in water may be associated with prolonged labour, and an increased used of oxytocin and epidural analgesia [6]. Women labouring at home may use water immersion for pain relief before the midwife arrives and may have used water immersion earlier in labour than women in an FMU or AMU. We lacked the data to explore these and other possible explanations further, but it is important to consider that a lower transfer rate in planned home births may not be the best outcome. The appropriate transfer rate for the home birth setting is unknown. However, the primary Birthplace analysis showed that in low risk women, the incidence of adverse perinatal outcomes in planned FMU and AMU births did not differ significantly from outcomes in planned OU births, but that for nulliparous women planned home birth was associated with a significantly increased risk of an adverse perinatal outcome [12]. We observed no significant difference in Apgar score less than seven at five minutes or in neonatal admissions between immersion and non-immersion in water for any of the settings, but our sample size was too small to investigate other less common and potentially more serious neonatal outcomes.

## Conclusions

For nulliparous women planning birth in a non-obstetric unit setting, immersion of water for pain relief is associated with a significantly lower risk of transfer before birth, a higher chance of a straightforward vaginal birth and a lower risk of intrapartum caesarean section. The benefits of immersion appear to be strongest in planned FMU births and weakest in planned home births. The findings of this large, observational study support a policy of offering immersion in water for pain relief to low risk healthy women with uncomplicated pregnancies [22], but the potential benefits and risks of immersion in water at home are less well established.

## Abbreviations

AMU: Alongside midwifery unit; CI: Confidence interval; FMU: Freestanding midwifery unit; OU: Obstetric unit; RR: Relative risk.

## Competing interests

The authors declare that they have no competing interests.

## Authors’ contributions

JH conceived the idea and developed the outline for this study. ML developed the analysis plan and conducted the analysis with advice and supervision from JH. ML and JH drafted the manuscript with input from all authors. All authors were involved in the interpretation of data, review and revision of the draft manuscript and approval of the final version.

## Pre-publication history

The pre-publication history for this paper can be accessed here:

http://www.biomedcentral.com/1471-2393/14/60/prepub

## Supplementary Material

Additional file 1: Figure S1 Study inclusion and exclusion flow chart. Click here for file

Additional file 2: Table S1 Restricted analysis of the association between immersion in water and risk of transfer. Click here for file

Additional file 3: Table S2 Association between immersion in water and neonatal outcomes. Click here for file
