# Supplementary material for: Immersion in water for pain relief and the risk of intrapartum transfer among low risk nulliparous women: secondary analysis of the Birthplace national prospective cohort study
Source: BMC Pregnancy Childbirth. 2014 Feb 6;14:60. doi: 10.1186/1471-2393-14-60 (PMC3922427; doi:10.1186/1471-2393-14-60)
Supplement: Additional file 1: Figure S1 — Study inclusion and exclusion flow chart. [file 1471-2393-14-60-S1.docx]

**Figure S1: Study inclusion and exclusion flow chart**

Original Birthplace cohort N=79,774

Nulliparous women, N= 35,234

Planned AMU, FMU, Home births N=18,805

Low risk at start of care in labour N= 16,702

‘Low risk’ N= 17,917

Multiparous women, 44,397

Parity not known, 143

Missing data on immersion in water, 125

Conditions “contraindicating” immersion in water at start of care in labour^*^, 1,215

‘Higher risk’ or risk status unknown, 888

Planned OU births, 16,429

Study population

Eligible low risk, nulliparous women planning a non OU birth

N= 16,577

AMU= 7,733

FMU= 4,831

Home= 4,013

*Prolonged rupture of membranes (>18 hours), meconium stained liquor, hypertension, abnormal vaginal bleeding, abnormal fetal heart rate, non-cephalic presentation.
