# Supplementary material for: Immersion in water for pain relief and the risk of intrapartum transfer among low risk nulliparous women: secondary analysis of the Birthplace national prospective cohort study
Source: BMC Pregnancy Childbirth. 2014 Feb 6;14:60. doi: 10.1186/1471-2393-14-60 (PMC3922427; doi:10.1186/1471-2393-14-60)
Supplement: Additional file 2: Table S1 — Restricted analysis of the association between immersion in water and risk of transfer. [file 1471-2393-14-60-S2.docx]

| **Supplementary table S1: Restricted analysis of the association between immersion in water and risk of transfer** | | | | | | | | | | |
| --- | --- | --- | --- | --- | --- | --- | --- | --- | --- | --- |
|  | Events | | Births | Weighted^1^ | | Unadjusted^1^ | | | Adjusted^1,2^ | |
|  | n | | n | % | (95% CI) | RR | | (95% CI) | RR | (95% CI) |
| **Transfer before birth (restricted sample^3^)** | | | | | | | | | | |
| **Home** | |  |  |  |  |  |  | |  |  |
| No immersion | | 526 | 1519 | 34.3 | (31.2–37.4) | 1 | - | | 1 | - |
| Immersion | | 564 | 1812 | 30.0 | (27.6–32.6) | 0.88 | (0.78–1.00) | | 0.85 | (0.75–0.96) |
| **FMU** | |  |  |  |  |  |  | |  |  |
| No immersion | | 617 | 1785 | 31.8 | (27.4–36.5) | 1 | - | | 1 | - |
| Immersion | | 550 | 2413 | 20.5 | (17.2–24.2) | 0.64 | (0.54–0.77) | | 0.60 | (0.50–0.72) |
| **AMU** | |  |  |  |  |  |  | |  |  |
| No immersion | | 1404 | 3910 | 35.5 | (32.1–39.0) | 1 | - | | 1 | - |
| Immersion | | 776 | 2670 | 29.3 | (25.4–33.4) | 0.83 | (0.72–0.95) | | 0.77 | (0.68–0.87) |
| ^1^ Weighted to adjust for clustering and each unit’s duration of participation  ^2^ Adjusted for maternal age, ethnic group, understanding of English, marital/partner status, index of multiple deprivation score quintile, and gestation (completed weeks).  ^3^ Excluding women for whom the decision to transfer was taken within the first 90 minutes of labour care, who gave birth within the first 90 minutes or for whom the time of birth or transfer was not known | | | | | | | | | | |
