# Supplementary material for: Immersion in water for pain relief and the risk of intrapartum transfer among low risk nulliparous women: secondary analysis of the Birthplace national prospective cohort study
Source: BMC Pregnancy Childbirth. 2014 Feb 6;14:60. doi: 10.1186/1471-2393-14-60 (PMC3922427; doi:10.1186/1471-2393-14-60)
Supplement: Additional file 3: Table S2 — Association between immersion in water and neonatal outcomes. [file 1471-2393-14-60-S3.docx]

| **Supplementary** **Table S2: Association between immersion in water and neonatal outcomes** | | | | | | | | |
| --- | --- | --- | --- | --- | --- | --- | --- | --- |
| Events | | Births | Weighted^1^ | | Unadjusted^1^ | | Adjusted^1,2^ | |
|  | n | n | % | (95% CI) | RR | (95% CI) | RR | (95% CI) |
| **Apgar Score below 7 at 5 minutes** | | | | | | | | |
| **Home** |  |  |  |  |  |  |  |  |
| No immersion | 23 | 1992 | 1.3 | (0.8–2.2) | 1 | - | 1 | - |
| Immersion | 31 | 2007 | 1.4 | (1.0–1.9) | 1.01 | (0.56–1.81) | 0.96 | (0.55–1.70) |
| **FMU** |  |  |  |  |  |  |  |  |
| No immersion | 23 | 2214 | 1.0 | (0.7–1.5) | 1 | - | 1 | - |
| Immersion | 27 | 2611 | 0.8 | (0.5–1.2) | 0.79 | (0.42–1.49) | 0.78 | (0.40–1.51) |
| **AMU** |  |  |  |  |  |  |  |  |
| No immersion | 54 | 4796 | 1.1 | (0.7–1.8) | 1 | - | 1 | - |
| Immersion | 24 | 2923 | 1.2 | (0.6–2.6) | 1.06 | (0.41–2.72) | 1.01 | (0.46–2.21) |
| **Neonatal admission** | | | | | | | | |
| **Home** |  |  |  |  |  |  |  |  |
| No immersion | 48 | 1986 | 2.6 | (1.9–3.6) | 1 | - | 1 | - |
| Immersion | 53 | 1998 | 2.5 | (1.9–3.2) | 0.94 | (0.61–1.44) | 0.89 | (0.58–1.36) |
| **FMU** |  |  |  |  |  |  |  |  |
| No immersion | 56 | 2213 | 2.4 | (1.7–3.2) | 1 | - | 1 | - |
| Immersion | 51 | 2614 | 1.7 | (1.2–2.6) | 0.74 | (0.49–1.12) | 0.68 | (0.43–1.08) |
| **AMU** |  |  |  |  |  |  |  |  |
| No immersion | 112 | 4763 | 2.5 | (1.9–3.3) | 1 | - | 1 | - |
| Immersion | 64 | 2907 | 2.5 | (1.7–3.5) | 0.98 | (0.64–1.51) | 0.94 | (0.62–1.43) |
| ^1^ Weighted to adjust for clustering and each unit’s duration of participation  ^2^ Adjusted for maternal age, ethnic group, understanding of English, marital/partner status, index of multiple deprivation score quintile, and gestation (completed weeks). | | | | | | | | |
